# Required displacement factors for evaluating and comparing climate impacts of intensive and extensive forestry in Germany

**DOI:** 10.1186/s13021-022-00216-8

**Published:** 2022-10-01

**Authors:** Buschbeck Christian, Pauliuk Stefan

**Affiliations:** Industrial Ecology Group, Faculty of Environment and Natural Resources, University of Freiburg, Tennenbacher Strasse 4, Freiburg im Breisgau, Germany

**Keywords:** Forest management, Wood utilization, Carbon storage, Carbon balance, Displacement factor, Substitution, Decarbonization

## Abstract

**Background:**

Forestry plays a major role in climate change mitigation. However, which intensity of logging is best suited for that task remains controversial. We contribute to the debate by quantitatively analyzing three different forest management scenarios in Germany—a baseline scenario which represents a continuation of current forest management practice as well as an intensive and an extensive logging scenario. We assess whether increased carbon storage in wood products and substitution of other emission-intensive materials can offset reduced carbon stocks in the forest due to increased harvesting. For that, we calculate annual required displacement factors (RDF)—a dimensionless quantity that indicates the minimal displacement factor (DF) so that intensive forestry outperforms extensive forestry from a climate perspective.

**Results:**

If the intensive forest management scenario is included in the comparison, the RDF starts off with relatively high values (1 to 1.5) but declines over time and eventually even reaches negative values. Comparing the extensive scenario to a baseline yields RDF values between 0.1 and 0.9 with a slightly increasing trend. Compared to RDFs, expected future DFs are too low to favour the intensive forestry scenario and too high to favour the extensive forestry scenario, during the first 25 years of the modeling period. However, towards the end of the modeling period, the relationship between DFs and RDF is turned around in both comparisons. In the comparison between intensive and extensive forest management RDF values are very similar to future DF trajectories.

**Conclusion:**

RDFs are a useful tool for comparing annual climate impacts of forest growth scenarios and can be used to benchmark material and energy substitution effects of wood. Our results indicate that the baseline scenario reflects an effective compromise between carbon stocks in the forest and carbon displacement by wood use. For a longer modeling period, however, this might not be the case. Which of the alternative scenarios would be best suited for climate change mitigation is heavily dependent on future DF trajectory. Hence, our findings highlight the necessity of robust projections of forest dynamics and industry decarbonization pathways.

**Supplementary Information:**

The online version contains supplementary material available at 10.1186/s13021-022-00216-8.

## Introduction

The question of how German forests should be managed in the future is highly topical [6]. In addition to the fulfillment of different ecosystem services (e.g., the regulation of water and nutrition cycles, the provision of timber, human recreation), the climate impact of forest management is of central importance, since their potential to act as carbon sink represents an effective tool for mitigating climate change. Accordingly, the German government has set its focus on the sink capacity of German forests in order to achieve carbon neutrality in the coming decades. For the carbon footprint of the land use, land use change and forestry sector (LULUCF) , ambitious targets have been set for future decades (− 25 mio t CO_2_-eq. in 2030, − 35 mio t CO_2_-eq. in 2040 [2]). Reducing emissions in the categories agriculture, grassland, wetland, and residential areas is one way to reduce the carbon footprint of this sector. However, the only categories within the sector that can act as major sinks are forestry and wooden products, through their ability to store carbon. According to a projection by the Federal Environment Agency (UBA), it is not possible to meet the sectoral targets by continuing current forest management practices [14]. New calculations based on more recent data come to the same conclusion, although the emission gap is less severe (3 to 17 Mt $CO_{2}$-eq. instead of 45) [22]. One option to meet the carbon sequestration goals of the German government would be to reduce logging, which in turn would cause higher greenhouse gas emissions in other sectors (e.g., the construction sector) or export countries due to the need to substitute the wood with other energy carriers and materials [2]. The assessment of climate benefits of forest management thus not only depends on actions and measures in the forest sector but also on the carbon intensity of energy supply and material production. Such cross-sectoral relationships highlight the necessity of consequential approaches (“how are the global environmental burdens affected by the production and use of the product?” [13]) in addition to the attributive approach (“what share of the global environmental burdens belongs to a product?” [13]) for assessing climate impacts of forest systems [10]. Many studies apply consequential approaches to assess whether intensive or extensive forest management is more beneficial for mitigating climate change [7, 15, 38], though the results are controversial.The main two reasons in favor of intensive forestry are that (1) an increase in wood utilization can offset decreased carbon stocks in the forest by storing more carbon in harvested wood products (HWP) and (2) by substituting more $CO_{2}$-intensive materials and services. [17, 18, 43]. Findings of other scholars support the opposite [26, 29, 39, 40]. This dissent can have several causes. For example, with regard to German forests, the controversy is related to the robustness of the carbon sequestration scenarios produced by different forest growth models used [1, 8]. The chosen time horizon can also have an impact: In a study from Gustavsson et al. [18], a very long time period of 200 years is analyzed. Here, climate impacts during the first decades of the modeling period promote extensive forestry because of higher carbon stock changes in the forest. Benefits of intensive forestry are only visible in the long run when the carbon sink in the extensive scenario is saturated. Studies that focus on shorter time horizons may ignore such long-term effects in the carbon balance [40]. Another dominant factor for divergent results in climate impact assessment is the choice and application of displacement factors (DF) [3]. They indicate how much carbon emissions would be avoided (substituted) by using wood instead of a non-wood alternative, and are defined as follows.

1 $$\begin{aligned} DF = \frac{GHG_{non-wood} - GHG_{Wood}}{WU_{Wood} - WU_{non-wood}} \end{aligned}$$

“where $GHG_{non-wood}$ and $GHG_{wood}$ are the GHG emissions resulting from the use of the non-wood and the wood alternatives, respectively, expressed in mass units of carbon (C) corresponding to the $CO_{2}$ equivalent of the emissions, and $WU_{wood}$ and $WU_{non-wood}$ are the amounts of wood used in the wood and non-wood alternatives, respectively, expressed in mass units of C contained in the wood” [37]. A vast variety of DF estimates exist in the literature, but there is a lack of coherence, transparency and robustness in those estimates [41]. Therefore we refrain from calculating climate impacts with fixed DFs in this study. Instead, DFs are treated as an uncertain and scenario-dependent variable. A method with a similar approach called required displacement factors (RDF), was already introduced by Seppälä et al. [39], and it can be used to compare climate impact assessments of different forest management scenarios. Because RDFs are solely calculated based on the forest dynamics and the HWP storage period, they are not subject to uncertainties arising from DF calculation but only to uncertainties stemming from forest growth modeling and HWP storage time.

### Required displacement factors for comparing the climate benefit of two forest management scenarios

RDFs are defined as the minimum DF that would be required for intensive forestry to climate-wise outperform extensive forestry [39]. They are used to compare different forest management scenarios and are based on the assumption that more raw wood can be used in an intensive forestry scenario compared to an extensive forestry scenario, resulting in larger substitution effects in the former. The only necessary input to calculate RDFs are parameters from forest growth modeling and the HWP lifetime because DFs are treated as exogenous parameter. By comparing calculated RDFs and DFs, a statement can be made about the climate impact of the different scenarios: If the average DF of wood use is higher than the RDF, the global warming potential (GWP) of the intensive scenario is lower than that of the extensive (and vice versa). In doing so, however, uncertainty of DFs would need to be addressed and discussed properly.

In a typical scenario comparison, the GWP of both scenarios is calculated based on forest carbon stocks, HWP carbon stocks, and substituted greenhouse gas (GHG) emissions. Forest carbon stocks can be derived from forest growth modelling and displaced emissions are calculated by multiplying DFs with the difference in wood used for a specific material or energy substitution option. For calculating HWP carbon stocks, different methods have been elaborated during the last decades. In 2006, the Intergovernmental Panel on Climate Change (IPCC) proposed three different approaches [33] from which the production approach was recommended for good practice a few years later [23]. In this approach, only wood products from domestically harvested wood are inventoried, ignoring changes in the wood product pool due to international trade [44]. Evidently, estimating HWP stocks is challenging and induces uncertainties, because the import and export of wood, as well as inherited emissions from the HWP stock before the modeling period, have to be dealt with [34, 44, 45]. In contrast to a country-specific carbon balance, where HWP carbon stocks are needed to correctly allocate climate impacts, the calculation of RDFs does not require them. Here, the uncertainties of HWP stock data are irrelevant for the assessment of the climate impacts of carbon storage in products.

So far, no RDFs have been computed for German forest management scenarios. RDF estimates exist for Finnish forests, with Seppälä et al. [39] calling for application of their method to other countries. We add to this method a refined approach for assessing the effect of temporal carbon storage in wood products and study how increasing the lifetime of wood products (through more sustainable wood use, such as cascade use [24]) affects the RDF.

### Goal and scope definition

In this work, we calculate RDFs for the management of German forests. We do so by evaluating published scenarios from established forest growth models. This consequential approach complements the attributive, sectoral approach for which GWP assessments are available. In calculating RDFs, we propose a new method that uses dynamic LCA (DLCA) [30] to quantify the climate implications of temporary storage of carbon within HWPs instead of the usual methodology that uses HWP carbon stocks. RDFs are calculated for different average wood product storage times to study the impact of this important technical parameter on the overall climate performance of different logging scenarios. Finally, the calculated RDFs are compared to the current average and future estimated DFs for different national climate policy scenarios. This comparison will test the assumption as to whether, from a climate perspective, higher storage and substitution effects can outweigh reductions in forest carbon stocks.

## Methods

### System definition

**Fig. 1:**
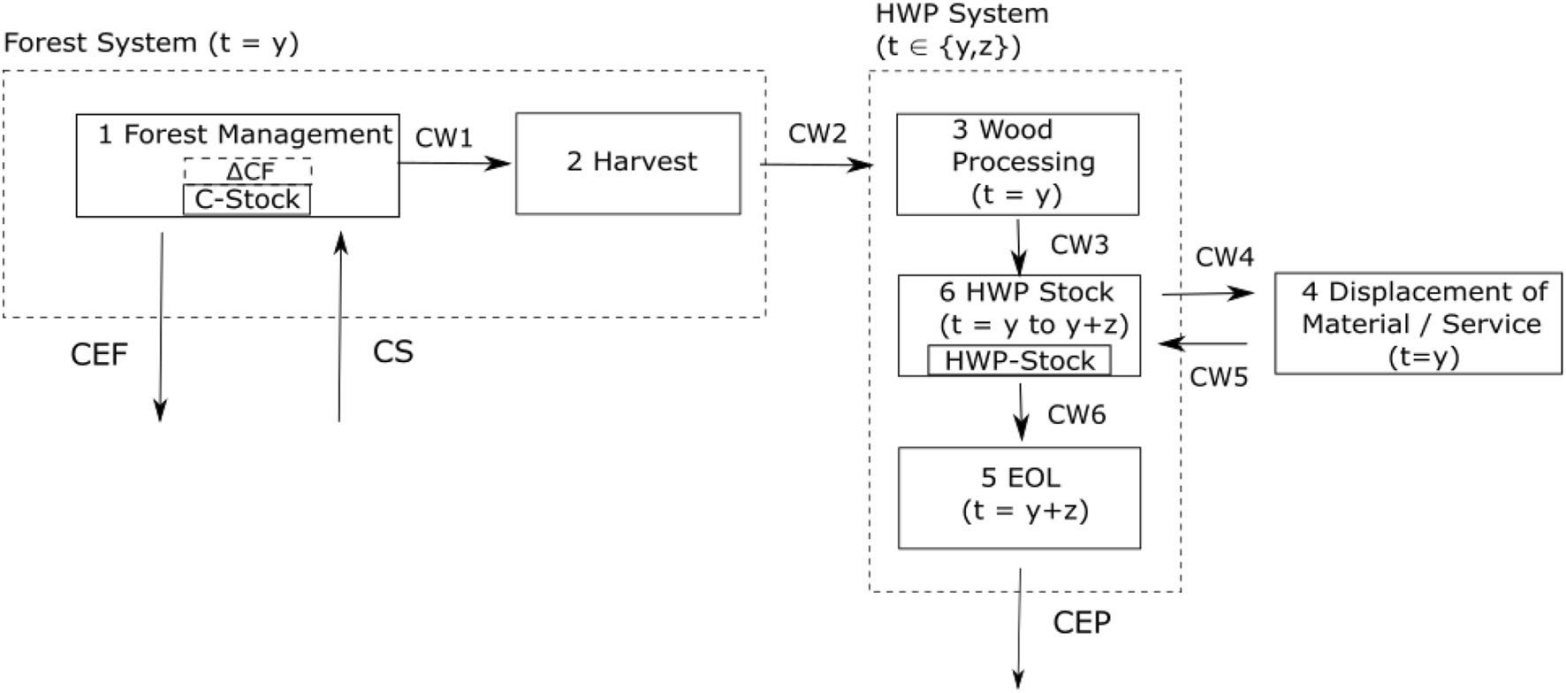
System definition for the forest- and HWP subsystems. CEF denotes the carbon emission of the forest and CS the carbon sequestration. CW1 to CW4 denote the carbon contained in wood and CEP the carbon emissions at the end of life (EOL) of the HWP

The forest-wood system is divided into two subsystems (Fig. 1). In the forest subsystem, carbon enters via sequestration (*CS*), and leaves the forest in form of emissions (*CEF*) (transpiration, decomposition of biomass, etc.) as well as in carbon stored in harvested wood (*CW*2). The sum of these three fluxes gives the carbon stock changes of the forest $\Delta CF$, via the mass balance for the forest management process. All three flows are quantified for any given time step within the modeling period (t=y). After harvest, the carbon sequestered in the wood enters the HWP subsystem. Due to wood utilization and material and energy substitution, GHG emission outside the system boundary are displaced or avoided. At the end of the HWP lifetime z, the carbon stored in the HWP is released as emission (*CEP*). The only difference in carbon uptake and release in the wood product subsystem is its timing (t=y and t=y+z, respectively). For this work, HWP does not only include material usage of wood but also energetic usages. The carbon fluxes shown are considered for annual time steps, i.e., annual carbon stock changes and annual extraction of biogenic carbon are accounted for.

### Required displacement factors with dynamic life cycle assessment

In accordance with the system definition (Fig. 1) the carbon balances for the two subsystems can be formulated. As convention for life cycle assessment (LCA), flows to the atmosphere have a positive sign, those from the atmosphere have negative sign. Accordingly, export of carbon from one product system to another has a positive, and import negative sign.

2 $$\begin{aligned} \begin{aligned} - CS + CEF + CW2 - CW2 + CW4 - CW5 + CEP = \Delta CATM \end{aligned} \end{aligned}$$

Where $\Delta CATM$ denotes for the carbon change in the atmosphere induced by forestry and wood utilization. The first three terms of equation 2 equal $\Delta CF$. The values for CW1to CW4 are both the same (*CW*). So we can simplify to

3 $$\begin{aligned} \Delta CF_{t=y} - CW_{t=y} + CW_{t=y+z} = \Delta CATM_{t \in \{y,y+z\} } \end{aligned}$$

The global warming potential *GWP* indicates the climate impact for the wood extraction in year *y* for a scenario *s*. $\Delta CF$ stands for the carbon stock change in the forest and *CW* for the carbon contained in the extracted wood. The subscript *t* gives the time of the emission and *z* represents the storage period in years.

The left part of equation 3 consists of several pulse emissions occurring at different times and can therefore be interpreted as the emissions profile of te system.When dealing with emission profiles, it is important to mention that timing plays a crucial role in assessing the climate effect of an emission. E.g., if two equal amounts of $CO_2$ are released to the atmosphere at two different points in time, they differ in their effect on climate change up to a given time horizon, because the earlier of the two had more time to heat up the atmosphere. Simple carbon- or GHG balances cannot capture this effect. In GWP however, timing can be included (with dynamic LCA [30]) because the GWP is always calculated for a certain time horizon. The most commom time horizon for GWP calculation is 100 years. However, in our case, forest dynamics are projected for several decades into the future and we consider storing the carbon in wood products for a couple of decades more. With a time horizon of only 100 years, substantial climate effects might occur beyond this relatively short period, and we therefore apply a time horizon of 500 years instead. With the modelling period in this study starting in 2012, this means that for the GWP calculation, the radiative forcing is always integrated up to the year 2512. In the emissions profile of a wood extraction, the storage of carbon in wood products is represented by the storage period *z*. Thus, by changing the storage period for wood products, the emissions profile changes but the time horizon up to which the effect on global warming is considered stays the same. We study how the uncertainty of the average storage period affects the RDF by calculating the RDF for a range of average storage periods. Burschel et al. [11] estimated the average storage time to be 1 and 65 years for energy wood and construction wood, respectively [35]. Frühwald et al. set the average storage time for construction wood in Germany to 75 years [16]. Therefore, the range for average storage periods used in this study lies between 1 and 75 years.

In the following section, the dynamic LCA method proposed by Levasseur [30] is briefly explained: First, dynamic characterization factors (DCF) are obtained (equation 4) by step-wise calculation of the cumulative radiative forcing from a factor for radiative efficiency ($\alpha$) and the impulse response function (*IRF*(*t*)), which describes the decay of a unit mass of a certain GHG in the atmosphere (here only $CO_{2}$ is considered). Then, the annual emissions $g_{y}$ are multiplied with their respective DCF and are summed up over the whole life cycle and again over each year to yield the cumulative time dependent impact on global warming $GWI_{cum}$ (equation 5). Lastly, by dividing $GWI_{cum}$ until a certain time horizon *TH* by the cumulative radiative forcing of a reference emission at time zero until *TH*, the total global warming potential (GWP) is obtained (equation 6).

4 $$\begin{aligned} DCF_{t}= & {} \int _{t-1}^{t} \alpha * IRF(t) \,dt \end{aligned}$$

5 $$\begin{aligned} GWI_{cum}(TH,g_y)= & {} \sum _{Y=0}^{TH} \sum _{y=0}^{Y} g_{y} * DCF_{Y-y} \end{aligned}$$

6 $$\begin{aligned} GWP_{dyn 500}(g_y)= & {} DLCA(g_y,TH=500) = \frac{GWI_{cum}(TH=500,g_y)}{\int _{t=0}^{TH} \alpha * IRF(t) \,dt} \end{aligned}$$

Up to this point, the forest carbon stock change and temporal storage of carbon in wood products were addressed. The climate impact of substitution (or carbon displacement via energy and material use of wood) is calculated by multiplying a DF with the amount of carbon entering the HWP system (which is CW).

7 $$\begin{aligned} C_{displaced} = CW * DF \end{aligned}$$

Because forest management is the main driver for forest carbon dynamics and wood is the main forest product, the climate impact of forest carbon stock changes can be fully allocated to the extracted wood [21]. The same applies to the climate impact of temporal carbon storage and substitution effects. In essence, all climate impacts induced by the wood extraction of one year can be allocated to this specific wood extraction (equation 8). By accumulating the climate impacts of all wood extractions to the same time horizon (500 years), comparability across different forest management and substitution scenarios is ensured.

8 $$\begin{aligned} GWP_{s,y} =DLCA(TH=500,g_y=(\Delta CF_{s,y,t=y} - CW_{s,y,t=y} + CW_{s,y,t=y+z} - CW_{s,y,t=y} * DF_{y})*\frac{44}{12}) \end{aligned}$$

In equation 8, the forest sink (or source) is represented by $\Delta CF_{s,y,t=y}$. The carbon accounting for the HWP (and by that, the storage of carbon in HWP) is represented by $- CW_{s,y,t=y} + CW_{s,y,t=y+z}$. The substitution effect is represented by $- CW_{s,y,t=y} * DF_{y}$ (negative sign because of displacement).

The sum of these effects, $\Delta CF_{s,y,t=y} - CW_{s,y,t=y} + CW_{s,y,t=y+z} - CW_{s,y,t=y} * DF_{y}$ therefore represents the emission profile for a wood extraction in year *y*. The DLCA function describes the application of dynamic LCA to a certain emission profile (equation 4 to 6). Since these are given in carbon masses, they must be multiplied by the fraction of the molar mass of carbon in the $CO_{2}$ molecule ($\frac{44}{12}$) to obtain the amount in kg $CO_{2}$.

Now, the goal is to find a value for DF for which the respective wood removals of a certain year achieve the same climate impact for both scenarios (definition of RDF). Higher DFs than this value would automatically result in the scenario for intensive forestry (IF) achieving a better climate impact than the scenario for extensive forestry (EF). A value DF* for which the following equation holds:

9 $$\begin{aligned} GWP_{s=IF,y} = GWP_{s=EF,y} \end{aligned}$$

is the required displacement factor (RDF). Hence, if Eq. 8 is set into Eq. 9, and the term is resolved for DF, the RDF can be obtained (for details, see Additional file 1):

10 $$\begin{aligned} \scriptstyle RDF_{y}= \frac{DLCA( \Delta CF_{IF,y,t=y} - \Delta CF_{EF,y,t=y} - CW_{IF,y,t=y} + CW_{EF,y,t=y} + CW_{IF,y,t=y+z} - CW_{EF,y,t=y+z})}{DLCA(-CW_{EF,y,t=y} + CW_{IF,y,t=y} )} \end{aligned}$$

**Fig. 2:**
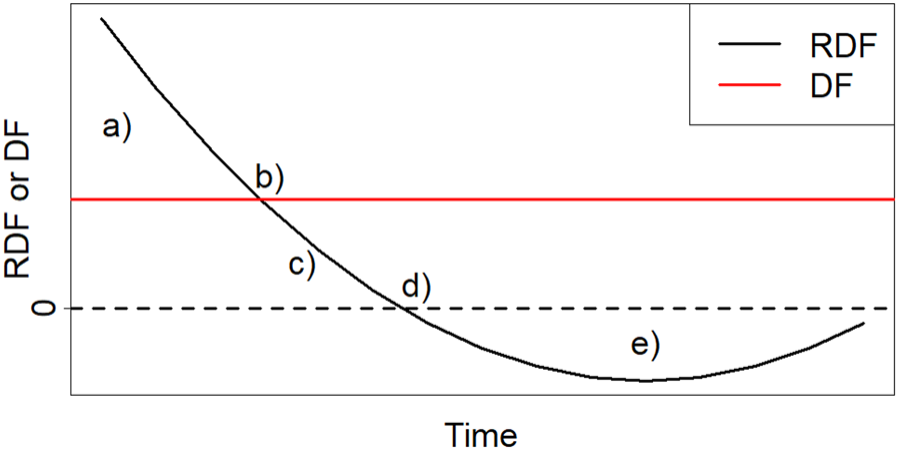
Stylistic examples of the relationship between DF and RDF curves. The implications of the shown cases (a-e) are described in the text

For $DF > RDF$, the wood removal of the intensive forestry scenario has a smaller global warming potential than that of extensive case. For $DF < RDF$, the wood removal of intensive forestry scenario has a larger global warming potential than that of extensive case. Following Insights can be derived by comparing DF and RDF curves:

- If $RDF> DF > 0$ (Fig. 2a): Substitution is needed for annual wood extractions in IF to achieve the same GWP as those in EF ($RDF > 0$). Stronger annual displacement in IF is too low to compensate differences in annual carbon stock changes ($RDF > DF$). $\rightarrow$ GWP of annual wood extractions is higher for IF than for EF
- If $RDF = DF > 0$ (Fig. 2b): Substitution is needed for annual wood extractions in IF to achieve the same GWP as those in EF ($RDF > 0$). Stronger annual displacement in IF can compensate differences in annual carbon stock changes ($RDF = DF$). $\rightarrow$ GWP of annual wood extractions is the same for IF and EF
- If $DF> RDF > 0$ (Fig. 2c): Substitution is needed for annual wood extractions in IF to achieve lower GWP as those in EF($RDF > 0$). Stronger annual displacement in IF is large enough to overcompensate differences in carbon stock changes ($DF > RDF$). $\rightarrow$ GWP of annual wood extractions is lower for IF than for EF
- If $DF > RDF = 0$ (Fig. 2d): No substitution is needed for annual wood extractions in IF to achieve the same GWP as those in EF ($RDF = 0$). Stronger annual displacement in IF is large enough to overcompensate differences in carbon stock changes ($DF > RDF$). $\rightarrow$ GWP of annual wood extractions is lower for IF than for EF
- If $DF> 0 > RDF$ (Fig. 2e): No substitution is needed for annual wood extractions in IF to achieve lower GWP as those in EF($RDF < 0$). Stronger annual displacement in IF is large enough to overcompensate differences in carbon stock changes ($DF > RDF$). $\rightarrow$ GWP of annual wood extractions is lower for IF than for EF

### Translating climate change mitigation scenarios into displacement factors

With the RDF approach proposed by Seppälä et al. [39], the forest climate assessment compares two forest management and wood product storage scenarios and is carried out independently of the actual substitution effect of the wood products. In order to make a general statement about which forest management scenario is better suited for climate change mitigation, it is necessary to estimate whether the average DFs of current and future wood utilization can reach calculated RDFs. A number of meta studies for average DFs are available from the literature [25, 28]. However, since the underlying system boundaries of the different DFs are not always consistent, the reported average DFs are subject to considerable uncertainties [41]. In order to minimize uncertainty, specific DFs for Germany were taken and mapped to the use type of German wood assortments [12, 27] (Table 1). Accordingly, a DF of 1.5 for wood as material substitution and a DF of 0.67 for wood used as energy carrier are used [27]. As there is no large-scale substitute for or alternative way of producing paper, the DF for pulp is set to 0 [39]. In a next step, the volume shares of the respective wood usage types were used as weight for calculating an average current DF for Germany. The resulting value of $DF_{avg}$ 1.13, is similar to the value for an average current DF used in the finnish study [39]. Although the DF values stem from a source from 2015, it is therefore assumed they still apply in the year 2022 as well.

For the estimation of future DFs, it is assumed that the shares of different uses of wood stay constant. DFs however, are bound to decline due to decarbonization of the energy supply and material production sectors [41]. Because estimating exact future DFs is very speculative, instead of using fixed values, conservative and optimistic estimates are established and used to define a range of possible DFs. Most studies in the literature estimate reduction potentials until the year 2050. However, because the German government recently formulated climate targets for carbon neutrality already in 2045, it is hereby assumed that these targets can be reached already in 2045 by increasing efforts in fighting climate change. Due to large reduction potentials in the energy and transport sector, it is assumed that GHG emissions caused by wood usage are negligibly small in 2045. Thus, the reduction in a material-specific DF is proportional to the reduction in GHG emissions of the material being substituted (Eq. 1).

Wood used as construction and non-construction material usually substitutes cement, concrete, metals and plastics [28]. By 2050, it is technically possible to reduce GHG emissions from the steel industry by 99.7%, GHG emissions from of metal production by 100%, and GHG emissions from the chemical industry by 98.5% [4]. In the cement industry, decarbonization is more challenging, because of process emissions in clinker production. How much these emissions can be reduced eventually is still under research. The German federal environmental agency estimates a reduction of 70% for the concrete sector until 2050 [4]. A case study for concrete production in China found that it should be possible to reduce emissions of concrete production by 50% until 2050 [42]. An up-to-date review on the matter also finds that a reduction of 50% is feasible [20]. Since it is not possible to predict for the future in which shares the different materials will be substituted, the highest and lowest reduction potential (100% and 50% respectively) of the mentioned industries are used to represent optimistic and conservative estimates for material substitution in future DFs.

For paper substitution, the DF is assumed to stay zero in both conservative and optimistic estimates.

The energy sector’s GHG emissions in 2050 could be reduced by between 40 and 98%, compared to current values, depending on the policy [32]. Accordingly, these reduction potentials were used to calculate conservative and optimistic estimates for future DFs

Table 1 shows the corresponding DFs (current and future) and wood utilization shares that we applied to estimate the respective weighted averages. To model the decrease from the current DF to the conservative or optimistic DF, a simple linear interpolation, starting from current values, is used.

**Table 1:** Shares of wood usages and estimated DFs

Consumer	Volume [1000 m]	Type of use	Share [%]	DF 2022	DF 2045	DF 2045
					Con.	Opt.
Sawmill industry	37274	Material	62.9	1.5	0.75	0
Wood-based panel industry	7957					
Veneer and plywood industry	720					
Pulp industry	6780	Paper	9.3	0	0	0
Biomass combustion plants (big)	486	Energetic	27.8	0.67	0.4	0.02
Biomass combustion plants (small)	664					
Private households	18612					
Pellet and briquette producers	597					
Weighted average				1.13	0.58	0

Values for wood Volumes were taken from [12] and Values for DF 2019 were taken from [27]. For future DFs reduction potentials were derived from [20, 32] resulting in a conservative (Con.) and an optimisitic (Opt.) estimate

### Using RDF and DF to calculate GWP differences

From the difference between DF and RDF, the difference in GWP of the annual wood extractions can be calculated: For $DF=RDF$, annual wood extractions for both scenarios have the same climate impact. Altering DF will only alter $CW_{s,y,t=y} * DF_{y}$. Hence, the difference between RDF and DF multiplied with the difference in annually extracted carbon, yields the annual GWP difference.

11 $$\begin{aligned} \Delta GWP_y = (RDF_y - DF_y) * (-CW_{EF,y,t=y} + CW_{IF,y,t=y} ) \end{aligned}$$

### Data

For German forests several forest growth models are available (e.g., SILVA, WEHAM (Wald Entwicklungs- und HolzAufkommens Modellierung) and FaBio (Forestry and Agriculture Biomass Model)). Regarding the projection of carbon stocks in extensive forestry scenarios, the results of WEHAM and FaBio deviate substantially, which led to an intense debate among forest growth modelers [1, 9]. For the purpose of this study, we use data provided by WEHAM because it is the most widely recognized of those Models. Its results are therefore well understood and the data is easily available. Additionally, the results of the WEHAM baseline scenario as well as two alternative scenarios already have been analysed in form of GHG balances [36]. Hence, the consequential approach of this work is meant to complement these already existing attributive results.

While the Baseline Scenario (here referred to as BL) assumes a continuation of current forestry practice, the two alternative scenarios are characterized by severe changes: In the wood preference scenario (WPS) (German acronym: HPS), the rotation period is reduced, which increases logging and the amount of extracted roundwood. Regarding species composition, in this scenario the fast-growing Douglas fir is increasingly planted instead of spruce and pine. In the nature preference scenario (NPS), the species composition is gradually altered to resemble the potential natural vegetation on the respective sites. This is achieved by shortening the rotation period of spruce and pine in the corresponding non-natural locations and replanting them with natural vegetation, which in turn increases the share of deciduous trees. Wood stocks in old aged stands and deadwood stocks are generally increased and more land is designated for nature conservation. The intensity of forest management increases in the following order across the three scenarios: NPS, BL, WPS.

**Fig. 3:**
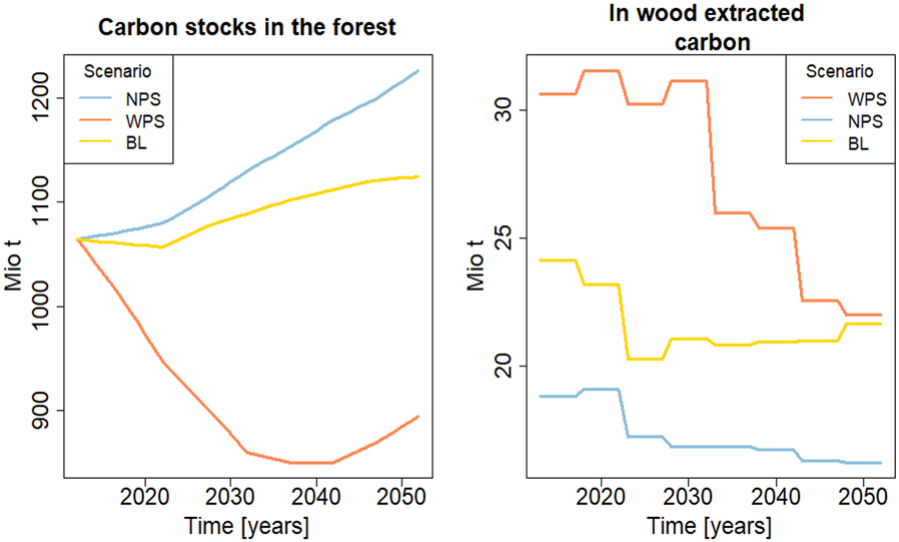
Forest carbon stocks (left) and carbon extracted in raw wood (right) for the WEHAM scenarios. Values were calculated from wood stocks and raw wood potential from [5] and [31] (see Additional file 1). WPS stands for wood harvest preference scenario. NPS stands for nature conservation preference scenario. BL stands for the baseline scneario

In order to calculate forest carbon stocks and the amount of annually extracted carbon, values for the development of wood stocks and raw wood potentials were taken [5, 31]. For a detailed description of the calculation can be found in the Additional file 1. Figure 3 shows the carbon stocks and the carbon extracted in raw wood for the three scenarios.

## Results

### Required displacement factors

**Fig. 4:**
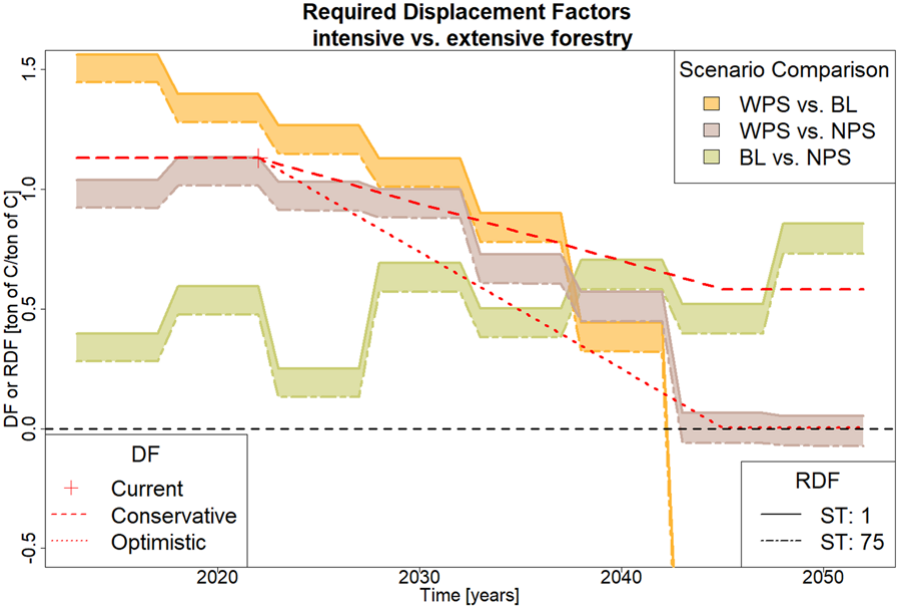
Evolution of required displacement factors (RDFs) with storage times (ST) ranging from 1 to 75 years. The current average displacement factor (DF) for wood utilization in Germany is indicated with a red cross and future DFs for different estimates of decarbonization (conservative, optimistic) are shown as red dotted lines

The evolution of RDFs over the modeling period is shown in Fig. 4. The curve describing the scenario comparison between BL and WPS starts with RDFs of about 1.5 and decreases over the decades, until it reaches negative values after 30 years (2042). In this comparison, RDF values are greater than DF values (each optimistic and conservative) for the first 25 years, then lie between both estimates, and finally, the RDF values drop well below DF values. In this comparison, BL reflects extensive forestry and WPS reflects intensive forestry. This means that first the annual wood extractions of BL have lower GWP than those of WPS, but later, it is vice versa. The curve describing the scenario comparison between BL and NPS starts with RDFs between 0.3 and 0.4, then oscillates over the decades, with a slightly increasing trend towards the end of the modelling period. RDFs do not change much and always lie between 0 and 1. In this comparison, RDF values are smaller than DFs for the first 25 years, then lie between the two estimates, and are greater than DFs for the last 5 years of the modeling period. Here, BL reflects intensive forestry and NPS reflects extensive forestry. This means that first, the annual wood extractions of BL have lower GWP than those of NPS, but later it is vice versa. The curve describing the scenario comparison between WPS and NPS starts with RDFs of slightly above 1, increase for about 10 years, but starts to decrease afterwards. This trend continues so that RDFs can even take on negative values (depending on the storage time) in the last years of the modelling period. In this comparison DF values are slightly above RDF values for the first 15 years. Afterwards, they mostly lie in between the DF range. This means that first, the annual wood extractions of WPS have lower GWP than those of NPS, but later, the climate benefit completely depends on the DF estimate.

The two curves containing the WPS show a similar trend. In both cases, relatively high DFs would be needed to offset the diminishing carbon stocks in the WPS (Fig. 3) resulting in relatively high RDFs. But over time, carbon stock changes of the WPS are similar (if not greater) compared to its respective counterpart. In combination with a greater amount of wood which can be stored and used to displace carbon, this scenario yields very small and even negative RDF values. The extremely low RDF values at the end of the modeling period for the comparison between WPS and BL arise because during that time, both scenarios have similar amounts of carbon extracted, but differ substantially in their carbon stock changes. This means, a very small difference in wood usage needs to compensate a large difference in carbon stocks. Because in this comparison, WPS is the intensive scenario but has greater values in carbon stock changes and extracted carbon, RDF values are negative.

The comparison between NPS and BL shows a relatively low RDF because in both scenarios carbon stocks are continuously increasing. However, in the BL the slope of carbon stocks declines over time. The same applies for the amount of extracted carbon, which leads to increasing RDF values.

Figure 4 also shows that increasing the average storage time of wood products can lower RDFs. However, high storage times, only feasible with long-lived construction wood, are needed for substantial effects: Increasing the storage time by 75 years causes a reduction in RDF of about 0.12.

### GWP differences between forest management scenarios under different industry decarbonization pathways

**Fig. 5:**
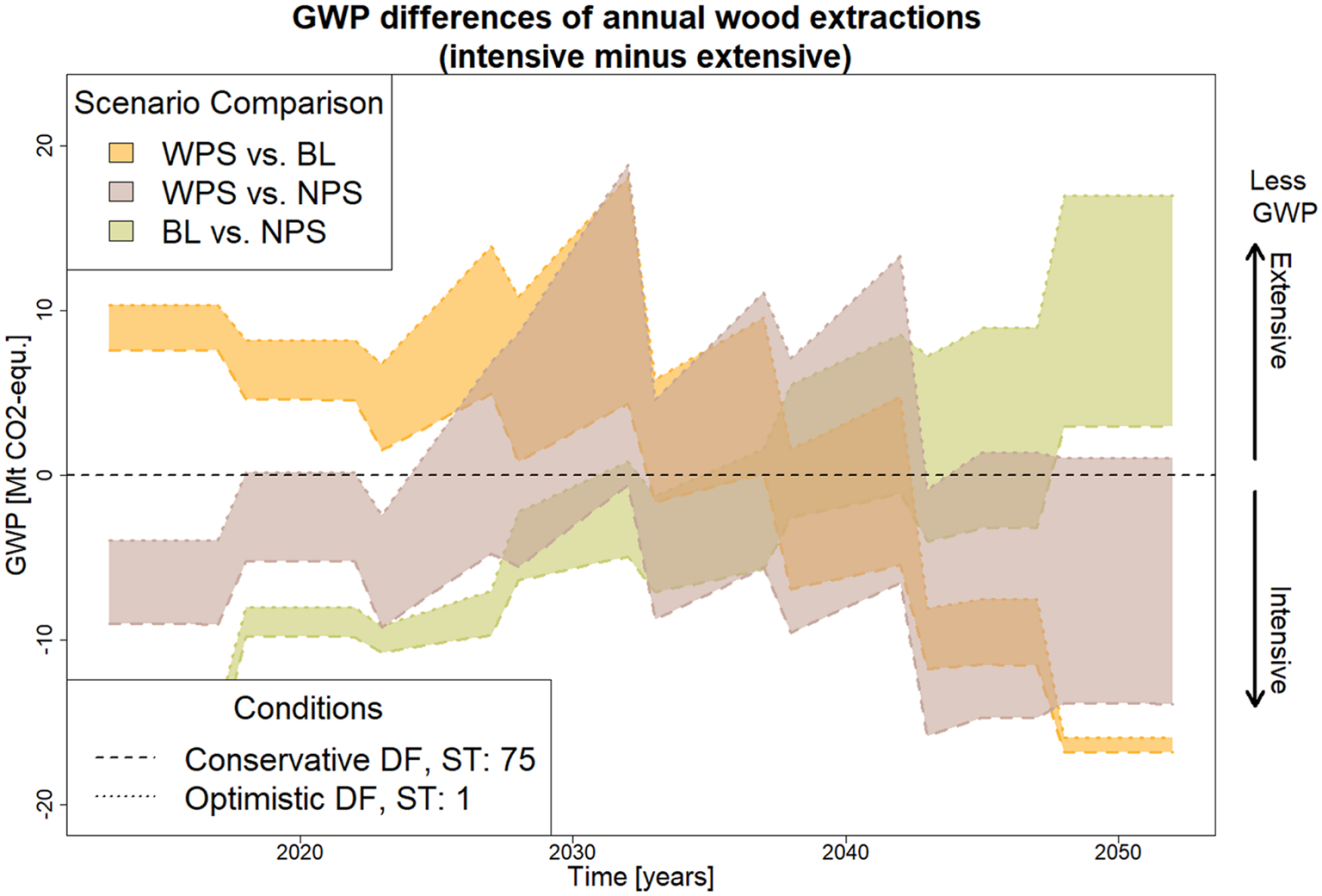
GWP-differences of annual wood extractions for conservative and optimistic DFs with different average storage times

By comparing DF and RDF, climate-related statements can be made about annual wood removals. In order to quantify the climate implications of the different scenarios, the annual GWP difference between wood removals in both scenarios can be calculated by multiplying the difference between DFs and RDFs with the difference in extracted carbon between both scenarios. The result is shown in Fig. 5. Since the GWP of EF is always subtracted from that of IF, positive values indicate lower GWP for EF and negative values indicate lower GWP for IF.

In the comparison between WPS and BL, the GWP differences in annual wood extractions show positive values during the first 25 years and negative values afterwards. For the comparison between NPS and BL it is vice versa. The GWP differences of annual wood extractions for the comparison between WPS and NPS do not follow a clear trend but roughly increase for around 20 years and decline afterwards. In this comparison the range of possible outcomes due to the wide range of possible DF estimates is the greatest. Often, whether the GWP difference is positive or negative solely depends on the DF estimate. This is due to the fact that RDFs in this comparison lie between the two DF boundary cases (red dotted lines in Fig. 4.

**Fig. 6:**
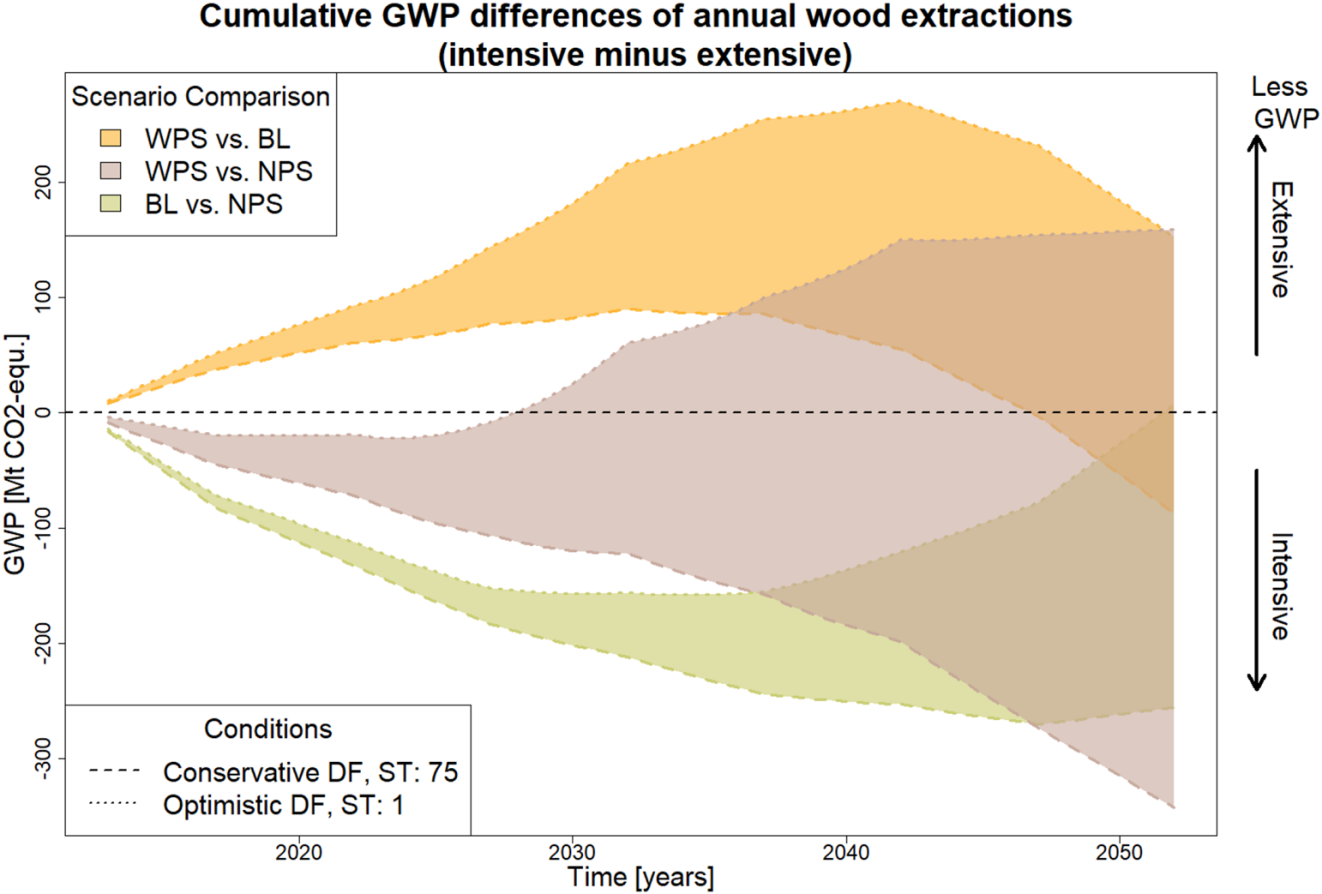
Cumulative GWP differences of annual wood extractions for conservative and optimistic DFs with different average storage times

A statement of the overall difference in climate effect between annual wood extractions of two forest management scenarios can only be made if their GWP differences are summed up. Since the GWP of wood removals in any given year is always calculated for the same time horizon (2512), they can simply be added together. Figure 6 shows the cumulative GWP differences. The value at the end of the period under study reflects the total GWP difference. The comparison between WPS and BL shows mostly positive values but tends to decline towards the end and even goes below zero. For the comparison between NPS and BL it is vice versa. Hence, in both comparisons, the BL has lower GWP in the first decades but higher GWP towards the end of the modelling period. In contrast to Fig. 4 and 5 the cumulative GWP differences can show which of those effects is more dominant: In total (i.e., at the end of the modeling period) the BL outperforms its counterparts in both comparisons (except for very high DF estimates and storage times in the comparison between WPS and BL). However, the trend of both curves indicates that with a longer modeling period, this might no longer be the case.

The comparison between WPS and NPS shows a huge range of possible values due to the wide range of possible future DFs. While both positive and negative values are possible, there is a tendency towards negative values. The total GWP difference (i.e., at the end of the modeling period) again is very much dependent on the DF estimate.

## Discussion

### Summary of the results

The evolution of RDFs is influenced by the interaction of carbon stocks and removals in the two compared scenarios. For the two comparisons where intensive forestry is represented by WPS, RDFs are relatively high (about 1 to 1.5) in the beginning of the modeling period but decline over the decades until 2050. This is mainly due to a radical transformation occurring in the WPS, where large carbon extractions through harvest lead to declining carbon stocks followed by a strong increase in carbon stocks due to subsequent re-growth. Increasing the storage time of timber products can reduce the RDF, but the effect is comparably small (increasing the storage period by 75 years would reduce RDFs by only about 0.12).

The comparison between NPS and BL produced a completely different RDF curve. Here, RDF values start off relatively low (between 0.3 and 0.4), tend to increase over time, but stay below 1. Looking at the carbon stocks in the forest, the carbon sink saturates for BL (carbon stock changes getting smaller), which is not the case in the NPS. The extracted carbon flows in the NPS and BL scenarios decline simultaneously. Thus, the diverging carbon stock changes lead to slightly increasing RDFs for this scenario pairing.

The calculated RDFs can be used to benchmark climate change mitigation strategies for the industry against climate change mitigation strategies regarding forests. Within the next decade, from a climate perspective:

- Intensive forestry according to WPS would be favorable if current average displacement factors were above 1.5
- Extensive forestry according to NPS would be favorable if current average displacement factors were below 0.1

From Fig. 4 it can be inferred that present substitution effects for wood products are large enough to compensate smaller carbon stocks in BL compared to NPS but are too small to be able to compensate smaller carbon stocks in WPS compared to BL ($RDF_{WPS-BL}> DF > RDF_{BL-NPS}$). Hence, the climate impact of current annual wood extraction is smallest for the baseline scenario. However, the trajectories of RDF and DF show that their relationship changes in the future. First, RDFs are smaller than DF for NPS against BL. At the end of the modeling period, RDF is becoming larger than the DF curve. For the comparison between WPS and BL it is vice versa. This means future annual wood extractions in BL will have higher climate impact compared to WPS and NPS. Throughout the modeling period, the early climate benefits of BL prevail, but the trend clearly indicates that a prolonged modeling period may shift the results towards a different conclusion 6

The comparison between WPS and NPS is dominated by the wide range of DF estimates as RDFs lie mostly between optimistic and conservative DF values. Hence, the question which of the two scenarios has lower climate impact is highly dependent on future decarbonization of the economy. This sheds light on the potential role of these two scenarios for climate change mitigation: Intensive forestry only performs better if the industry sector cannot be decarbonised properly by 2045. Therefore, WPS can be seen as a back-up plan for a future where industry decarbonization targets are not met. For extensive forestry to perform better than intensive forestry, ambitious industry carbon neutrality goals have to be largely met. Thus, the role of forest management as a climate change mitigation strategy does not solely depend on forest growth dynamics but also on the climate performance of other sectors, especially material production and energy supply. Decision makers need to be aware of this crucial cross-sector linkage and develop strategy bundles that robustly enable both forestry and industry to reach their climate goals.

### Limitations, model and data uncertainty and robustness of our central claims

It could be shown that RDFs can be a tool for investigating the relationship between different forest growth scenarios and are useful for benchmarking DFs. Yet, due to their comparative nature and the unit of measurement, they are not directly suitable to assess the overall climate performance of a certain scenario. Instead, climate impacts were calculated via GWP differences.

For all results presented here, substantial uncertainties have to be acknowledged and taken into account. RDFs were calculated from relatively old model results. Since the base scenario of the WEHAM model overestimated wood removals in recent years [22], it is reasonable to assume that similar discrepancies can be found in the alternative WEHAM scenarios as well. Also, changing forest growth dynamics and frequency of disturbances (drought, storm etc.) due to a changing climate were not considered when building the alternative WEHAM scenarios. Disturbances such as drought, windthrow or beetle infestation have the potential to reduce the sink effect of forests, either because damaged wood decomposes or because it needs to be extracted prematurely [22]. Assessing which forestry scenario, intensive or extensive, is influenced more by these calamities requires a new generation of forest growth models that include climate feedback (e.g. [19]).

In contrast, the uncertainty and bias of the DF trajectories may falsely enhance the climate performance of intensive forestry, as DFs tend to be systematically overestimated [29]. This is because a) the DF calculations usually assume that biomass is carbon neutral and b) the demand of the substituted material is assumed to be effectively reduced [29]. However, it is plausible and more likely that rebound effects from the additional supply of wood-based energy and materials occur, which means that current DF trajectories may be overestimated to some extent. If DFs are systematically overestimated, it becomes less likely that they can reach RDF levels, which in turn increases the climate advantage of extensive forestry.

Next to this bias, aggregating values for DFs across different materials and sectors adds large uncertainties [28]. Additional uncertainties arise when estimating future DFs. Here, a range of possible future target values and curves between current and future target values need to be defined, since the exact trajectory of future industry emissions is very speculative at this point.

### Comparison to the literature

The RDF results presented here can be compared to those in [39]. For Finnish forests, the resulting RDFs are larger than the average current DF. For Germany, that only applies when WPS and BL are compared. But the trend in Finnish RDFs resembles that of our comparison between BL and NPS, as both tend to increase over time. Differences between our results and those obtained by [39] may stem from the underlying forest growth scenarios.

Due to the short modeling period of 40 years, the results obtained here are only part of a bigger picture of future forest development. For example, in a study from Gustavsson et al. [18] initial high emissions of intensive forestry were compensated after about 40 years so that in the long run, it outperformed the extensive forestry scenario. Such trends can of course not be captured with the relatively short modeling period of this study, which is given by the time horizon of the WEHAM scenarios. Actually, the trend in cumulative GWP towards the end of the modelling period 6 suggests that the total climate impact will change substantially with a prolonged modeling period.

## Conclusion

With the help of required displacement factors (RDF), the consequential aspect of the climate impact of different forest management scenarios was assessed. Temporal storage of carbon in wood products was incorporated with dynamic LCA, so that the uncertainty arising from HWP stock estimation was eliminated. Still, the storage time has a comparably small impact on the required displacement factors.

The evolution of required displacement factors and displacement factors showed that in the short to mid-term, the baseline scenario reflects a good compromise between carbon stocks in the forest and carbon displaced by wood usage. But towards the end of the modeling period, annual wood extractions in baseline scenario have higher climate impact than those in the other scenarios. The question of whether the nature preference scenario or the wood preference scenario performs better in terms of climate change mitigation thus heavily depends on the future trajectory of displacement factors (i.e. the speed and intensity of industry decarbonization).

The scenario which is overall best suited for climate change mitigation cannot be inferred from RDF curves alone. By calculating cumulative GWP differences of annual wood extractions, it was confirmed that climate benefits in the baseline scenario prevail within the modeling period. However, the curves’ trend suggest that a prolonged modeling period would yield different results.

Although the direct policy implications of our analysis are limited due to severe uncertainties, we consider RDFs as a powerful tool for future research to gain insight in forest dynamics and to benchmark the performance of possible substitutions. Some of the named uncertainties will soon be alleviated: With the upcoming fourth national forest inventory, more recent data on German forest carbon flows and stocks will become available. In combination with forest growth modeling that takes climate change into account and with more reliable data to model possible decarbonization pathways, the robustness of the results will increase.

## Supplementary Information

**Additional file 1: Table S1.** Wood and carbon stocks in living trees in BL.**Table S2.** Wood and carbon stocks in living trees in WPS. **Table S3.** Wood and carbon stocks in living trees in NPS. **Table S4.** Deadwood stocks with the corresponding carbon masses for WPS and NPS. **Table S5.** Total carbon stocks in the forest. **Table S6.** Conversion factors for carbon in different tree species groups. **Table S7.** Wood and carbon volumes extracted in raw wood of the BL. **Table S8.** Wood and carbon volumes extracted in raw wood of the WPS. **Figure S1.** Wood stocks in BL and NPS from BMEL (2016) and Oehmichen et al. (2018). **Figure S2.** Raw wood extractions in BL (from BMEL (2016)). **Table S9.** Wood and carbon volumes extracted in raw wood of the NPS. **Figure S3.** Required displacement factors in a thought experiment where a change in forest management starts in 2032. **Figure S4.** Cumulative GWP differences in a thought experiment where a change in forest management starts in 2032.

## Data Availability

All data generated or analysed during this study are included in this published article and its additional information files. The model code is released under a permissive license on https://github.com/christianbuschbeck/RDF-Germany.git.

## Abbreviations

BL: Baseline scenario
$\Delta$ CATM: Difference of carbon in the atmosphere
$\Delta$ CF: Difference of carbon in the forest
CS: Sequestered carbon
CEF: Carbon emission of the forest
CW: Carbon stored in wood
CEP: Carbon emission of the product
DCF: Dynamic characterization factor
DF: Displacement factor
DLCA: Dynamic life cycle assessment
EF: Extensive forestry
EOL: End of life
FaBio: Forestry and Agriculture Biomass Model
FaBio: Forestry and Agriculture Biomass Model
GHG: Greenhouse gas
GWP: Global warming potential
HWP: Harvested wood product
IF: Intensive forestry
IPCC: Intergovernmental Panel on Climate Change
IRF: Impulse response function
LCA: Life cycle assessment
LULUCF: Land use, land use change and forestry
NPS: Nature conservation preference scenario
RDF: Required displacement factor
UBA: Federal Environmental Agency (German: Umweltbundesamt)
WEHAM: Wald Entwicklungs- und HolzAufkommens Modellierung
WPS: Wood preference scenario
