# Supplementary material for: Required displacement factors for evaluating and comparing climate impacts of intensive and extensive forestry in Germany
Source: Carbon Balance Manag. 2022 Oct 1;17:14. doi: 10.1186/s13021-022-00216-8 (PMC9526925; doi:10.1186/s13021-022-00216-8)
Supplement: Supplementary file 1 — Additional file 1: Table S1. Wood and carbon stocks in living trees in BL.Table S2. Wood and carbon stocks in living trees in WPS. Table S3. Wood and carbon stocks in living trees in NPS. Table S4. Deadwood stocks with the corresponding carbon masses for WPS and NPS. Table S5. Total carbon stocks in the forest. Table S6. Conversion factors for carbon in different tree species groups. Table S7. Wood and carbon volumes extracted in raw wood of the BL. Table S8. Wood and carbon volumes extracted in raw wood of the WPS. Figure S1. Wood stocks in BL and NPS from BMEL (2016) and Oehmichen et al. (2018). Figure S2. Raw wood extractions in BL (from BMEL (2016)). Table S9. Wood and carbon volumes extracted in raw wood of the NPS. Figure S3. Required displacement factors in a thought experiment where a change in forest management starts in 2032. Figure S4. Cumulative GWP differences in a thought experiment where a change in forest management starts in 2032. [file 13021_2022_216_MOESM1_ESM.pdf]

Additional information for ”Required displacement factors for evaluating and comparing climate impacts of intensive and extensive forestry in Germany”

September 29, 2022

1 Calculating carbon stocks in the forest

For this study, carbon stocks in the forest, are made up of two compartments: Carbon stocks in living trees and carbon stocks in deadwood. Carbon stocks in the soil and in the herbaceous and shrub layer were considered constant here and were therefore neglected.

1.1 Carbon in living trees

For the calculation of carbon stocks in living trees, wood stocks were taken from Oehmichen et al. (2018). All subsequent references to figures in this section therefore refer to this study. The same values were used as the starting point of wood stock development for both scenarios: 1.42 billion m^3 of hardwood and 2.24 billion m^3 of softwood (Figure 5 and Figure 22, respectively). For the WPS, it was then possible to use the documented values for wood stock change (Figure 6).
For the NPS, stock changes were not documented. Only the rounded values for wood stocks were available (Figure 22). In order to obtain exact values for the stock changes the data was retrieved from Figure 22 via the WebPlotDigitizer (https://automeris.io/WebPlotDigitizer/). For the BL, stock changes were retrieved from Figures 18, 24, 27, 30 in BMEL (2016) via the WebPlotDigitizer (https://automeris.io/WebPlotDigitizer/). Because all values were only available in aggregate form (5-year values), the changes in stock were divided equally among 1-year values.

In order to account for the densities of the different woods when calculating carbon stocks, different factors were used for the carbon contents of the respective wood types. The carbon contents of hardwood and softwood were calculated as the mean of the carbon contents of beech and oak, and spruce and pine, respectively. This ensures consistency between the carbon stock in the forest and the carbon extracted in raw wood. Table S6 shows these densities and resulting values for carbon content

Table S1: Wood and carbon stocks in living trees in **BL**. Both variables are given for hardwoods and conifers.

	Softwood		Hardwood		Total
C-Factor [kg/m^3]	247.5		340		-
Year	Wood mio m^3	C mio kg	Wood mio m^3	C mio kg	C mio kg
2013	2240.00	554400.00	1420.00	482800.00	1037200.00
2014	2242.67	555061.56	1415.83	481381.39	1036442.94
2015	2245.35	555723.11	1411.66	479962.77	1035685.89
2016	2248.02	556384.67	1407.48	478544.16	1034928.83
2017	2250.69	557046.23	1403.31	477125.55	1034171.77
2018	2253.36	557707.78	1399.14	475706.94	1033414.72
2019	2253.74	557800.03	1396.79	474908.31	1032708.34
2020	2254.11	557892.28	1394.44	474109.68	1032001.96
2021	2254.48	557984.52	1392.09	473311.06	1031295.58
2022	2254.86	558076.77	1389.74	472512.43	1030589.20
2023	2255.23	558169.02	1387.39	471713.80	1029882.82
2024	2262.42	559948.57	1393.99	473956.73	1033905.30
2025	2269.61	561728.12	1400.59	476199.66	1037927.78
2026	2276.80	563507.67	1407.18	478442.58	1041950.25
2027	2283.99	565287.22	1413.78	480685.51	1045972.73
2028	2291.18	567066.78	1420.38	482928.43	1049995.21
2029	2295.93	568242.08	1424.35	484278.16	1052520.24
2030	2300.68	569417.39	1428.32	485627.88	1055045.28
2031	2305.43	570592.70	1432.29	486977.61	1057570.31
2032	2310.17	571768.01	1436.26	488327.34	1060095.34
2033	2314.92	572943.32	1440.23	489677.06	1062620.38
2034	2319.07	573969.61	1445.28	491394.57	1065364.18
2035	2323.22	574995.90	1450.33	493112.09	1068107.99
2036	2327.36	576022.19	1455.38	494829.60	1070851.79
2037	2331.51	577048.48	1460.43	496547.12	1073595.60
2038	2335.66	578074.77	1465.48	498264.63	1076339.40
2039	2337.80	578604.73	1469.66	499683.24	1078287.97
2040	2339.94	579134.68	1473.83	501101.85	1080236.54
2041	2342.08	579664.64	1478.00	502520.47	1082185.10
2042	2344.22	580194.59	1482.17	503939.08	1084133.67
2043	2346.36	580724.55	1486.35	505357.69	1086082.24
2044	2348.40	581229.81	1490.13	506644.37	1087874.18
2045	2350.44	581735.08	1493.91	507931.04	1089666.12
2046	2352.49	582240.34	1497.70	509217.72	1091458.06
2047	2354.53	582745.61	1501.48	510504.40	1093250.00
2048	2356.57	583250.87	1505.27	511791.07	1095041.94
2049	2354.26	582680.25	1509.02	513066.07	1095746.32
2050	2351.96	582109.63	1512.77	514341.07	1096450.70
2051	2349.65	581539.01	1516.52	515616.07	1097155.08
2052	2347.35	580968.38	1520.27	516891.07	1097859.46

Table S2: Wood and carbon stocks in living trees in **WPS**. Both variables are given for hardwoods and conifers, as well as the forest in total.

	Softwood		Hardwood		Total
C-Factor [kg/m^3]	247.5		340		-
Year	Wood mio m^3	C mio kg	Wood mio m^3	C mio kg	C mio kg
2013	2240.00	554400.00	1420.00	482800.00	1037200.00
2014	2222.00	549945.00	1400.80	476272.00	1026217.00
2015	2204.00	545490.00	1381.60	469744.00	1015234.00
2016	2186.00	541035.00	1362.40	463216.00	1004251.00
2017	2168.00	536580.00	1343.20	456688.00	993268.00
2018	2150.00	532125.00	1324.00	450160.00	982285.00
2019	2125.00	525937.50	1305.60	443904.00	969841.50
2020	2100.00	519750.00	1287.20	437648.00	957398.00
2021	2075.00	513562.50	1268.80	431392.00	944954.50
2022	2050.00	507375.00	1250.40	425136.00	932511.00
2023	2025.00	501187.50	1232.00	418880.00	920067.50
2024	2005.40	496336.50	1220.80	415072.00	911408.50
2025	1985.80	491485.50	1209.60	411264.00	902749.50
2026	1966.20	486634.50	1198.40	407456.00	894090.50
2027	1946.60	481783.50	1187.20	403648.00	885431.50
2028	1927.00	476932.50	1176.00	399840.00	876772.50
2029	1905.00	471487.50	1165.80	396372.00	867859.50
2030	1883.00	466042.50	1155.60	392904.00	858946.50
2031	1861.00	460597.50	1145.40	389436.00	850033.50
2032	1839.00	455152.50	1135.20	385968.00	841120.50
2033	1817.00	449707.50	1125.00	382500.00	832207.50
2034	1811.40	448321.50	1123.40	381956.00	830277.50
2035	1805.80	446935.50	1121.80	381412.00	828347.50
2036	1800.20	445549.50	1120.20	380868.00	826417.50
2037	1794.60	444163.50	1118.60	380324.00	824487.50
2038	1789.00	442777.50	1117.00	379780.00	822557.50
2039	1789.40	442876.50	1116.60	379644.00	822520.50
2040	1789.80	442975.50	1116.20	379508.00	822483.50
2041	1790.20	443074.50	1115.80	379372.00	822446.50
2042	1790.60	443173.50	1115.40	379236.00	822409.50
2043	1791.00	443272.50	1115.00	379100.00	822372.50
2044	1801.20	445797.00	1118.80	380392.00	826189.00
2045	1811.40	448321.50	1122.60	381684.00	830005.50
2046	1821.60	450846.00	1126.40	382976.00	833822.00
2047	1831.80	453370.50	1130.20	384268.00	837638.50
2048	1842.00	455895.00	1134.00	385560.00	841455.00
2049	1855.40	459211.50	1139.20	387328.00	846539.50
2050	1868.80	462528.00	1144.40	389096.00	851624.00
2051	1882.20	465844.50	1149.60	390864.00	856708.50
2052	1895.60	469161.00	1154.80	392632.00	861793.00

Table S3: Wood and carbon stocks in living trees in **NPS**. Both variables are given for hardwoods and conifers.

	Softwood		Hardwood		Total
C-Factor [kg/m^3]	247.5		340		-
Year	Wood mio m^3	C mio kg	Wood mio m^3	C mio kg	C mio kg
2013	2240.00	554400.00	1420.00	482800.00	1037200.00
2014	2236.00	553410.00	1424.00	484160.00	1037570.00
2015	2232.00	552420.00	1428.00	485520.00	1037940.00
2016	2228.00	551430.00	1432.00	486880.00	1038310.00
2017	2224.00	550440.00	1436.00	488240.00	1038680.00
2018	2220.00	549450.00	1440.00	489600.00	1039050.00
2019	2212.00	547470.00	1448.00	492320.00	1039790.00
2020	2204.00	545490.00	1456.00	495040.00	1040530.00
2021	2196.00	543510.00	1464.00	497760.00	1041270.00
2022	2188.00	541530.00	1472.00	500480.00	1042010.00
2023	2180.00	539550.00	1480.00	503200.00	1042750.00
2024	2176.00	538560.00	1494.00	507960.00	1046520.00
2025	2172.00	537570.00	1508.00	512720.00	1050290.00
2026	2168.00	536580.00	1522.00	517480.00	1054060.00
2027	2164.00	535590.00	1536.00	522240.00	1057830.00
2028	2160.00	534600.00	1550.00	527000.00	1061600.00
2029	2156.00	533610.00	1566.00	532440.00	1066050.00
2030	2152.00	532620.00	1582.00	537880.00	1070500.00
2031	2148.00	531630.00	1598.00	543320.00	1074950.00
2032	2144.00	530640.00	1614.00	548760.00	1079400.00
2033	2140.00	529650.00	1630.00	554200.00	1083850.00
2034	2136.00	528660.00	1644.00	558960.00	1087620.00
2035	2132.00	527670.00	1658.00	563720.00	1091390.00
2036	2128.00	526680.00	1672.00	568480.00	1095160.00
2037	2124.00	525690.00	1686.00	573240.00	1098930.00
2038	2120.00	524700.00	1700.00	578000.00	1102700.00
2039	2114.00	523215.00	1716.00	583440.00	1106655.00
2040	2108.00	521730.00	1732.00	588880.00	1110610.00
2041	2102.00	520245.00	1748.00	594320.00	1114565.00
2042	2096.00	518760.00	1764.00	599760.00	1118520.00
2043	2090.00	517275.00	1780.00	605200.00	1122475.00
2044	2084.00	515790.00	1794.00	609960.00	1125750.00
2045	2078.00	514305.00	1808.00	614720.00	1129025.00
2046	2072.00	512820.00	1822.00	619480.00	1132300.00
2047	2066.00	511335.00	1836.00	624240.00	1135575.00
2048	2060.00	509850.00	1850.00	629000.00	1138850.00
2049	2056.00	508860.00	1866.00	634440.00	1143300.00
2050	2052.00	507870.00	1882.00	639880.00	1147750.00
2051	2048.00	506880.00	1898.00	645320.00	1152200.00
2052	2044.00	505890.00	1914.00	650760.00	1156650.00

1.2 Carbon in dead wood

According to Oehmichen et al. (2018), in the WPS, the deadwood stock remains constant at 14.6 $\frac{m^3}{ha}$. In the NPS, deadwood stock is projected to increase to 35 $\frac{m^3}{ha}$ within the study period. This increase is modeled by an annual deadwood replenishment. Since the deadwood supply increases linearly from 14.6 $\frac{m^3}{ha}$ to 35 $\frac{m^3}{ha}$ within 40 years, it is possible to interpolate between the two values for this period. Thereupon, the stocks per hectare can be multiplied by the total area of the forest to obtain the total deadwood stock in the forest. By further multiplying by the proportion of carbon in deadwood, the total carbon in deadwood in the forest is obtained. The carbon fraction of deadwood was calculated from 2017 greenhouse gas inventory data:

$$CarbonContentDeadwood = \frac{C_{dw}}{V_{dw} * A}$$

Where C_{dw} denotes the carbon in deadwood, V_{dw} denote the carbon stock per hectare, and A denote the area of deadwood considered in the greenhouse gas inventory. Using the appropriate values, we obtain:

$$CarbonContentDeadwood = \frac{33637225000kg}{22.2\frac{m^3}{ha} * 8598490ha} = 176.2\frac{kg}{m^3}$$

Table S4: Deadwood stocks with the corresponding carbon masses for WPS and NPS. For the WPS, deadwood stocks per hectare remain constant, while for the NPS they steadily increase. The increase in the NPS as well as the forest area with deadwood was taken from Oehmichen et al. (2018). The average carbon content (C-factor) was obtained from the 2017 greenhouse inventory.

Area	10620000 ha								
C-Factor	176.2 $\frac{kg}{m^3}$								
	BL			WPS			NPS		
	Deadwoodstock		C-Stock	Deadwoodstock		C-Stock	Deadwoodstock		C-Stock
Year	$\frac{m^3}{ha}$	mio m^3	mio kg	$\frac{m^3}{ha}$	mio m^3	mio kg	$\frac{m^3}{ha}$	mio m^3	mio kg
2013	14.60	155.05	27322.62	14.60	155.05	27322.62	14.60	155.05	27322.62
2014	14.60	155.05	27322.62	14.60	155.05	27322.62	15.12	160.61	28301.51
2015	14.60	155.05	27322.62	14.60	155.05	27322.62	15.65	166.16	29280.41
2016	14.60	155.05	27322.62	14.60	155.05	27322.62	16.17	171.72	30259.30
2017	14.60	155.05	27322.62	14.60	155.05	27322.62	16.69	177.27	31238.19
2018	14.60	155.05	27322.62	14.60	155.05	27322.62	17.22	182.83	32217.08
2019	14.60	155.05	27322.62	14.60	155.05	27322.62	17.74	188.38	33195.98
2020	14.60	155.05	27322.62	14.60	155.05	27322.62	18.26	193.94	34174.87
2021	14.60	155.05	27322.62	14.60	155.05	27322.62	18.78	199.49	35153.76
2022	14.60	155.05	27322.62	14.60	155.05	27322.62	19.31	205.05	36132.66
2023	14.60	155.05	27322.62	14.60	155.05	27322.62	19.83	210.60	37111.55
2024	14.60	155.05	27322.62	14.60	155.05	27322.62	20.35	216.16	38090.44
2025	14.60	155.05	27322.62	14.60	155.05	27322.62	20.88	221.71	39069.33
2026	14.60	155.05	27322.62	14.60	155.05	27322.62	21.40	227.27	40048.23
2027	14.60	155.05	27322.62	14.60	155.05	27322.62	21.92	232.82	41027.12
2028	14.60	155.05	27322.62	14.60	155.05	27322.62	22.45	238.38	42006.01
2029	14.60	155.05	27322.62	14.60	155.05	27322.62	22.97	243.93	42984.90
2030	14.60	155.05	27322.62	14.60	155.05	27322.62	23.49	249.49	43963.80
2031	14.60	155.05	27322.62	14.60	155.05	27322.62	24.02	255.04	44942.69
2032	14.60	155.05	27322.62	14.60	155.05	27322.62	24.54	260.60	45921.58
2033	14.60	155.05	27322.62	14.60	155.05	27322.62	25.06	266.15	46900.47
2034	14.60	155.05	27322.62	14.60	155.05	27322.62	25.58	271.71	47879.37
2035	14.60	155.05	27322.62	14.60	155.05	27322.62	26.11	277.26	48858.26
2036	14.60	155.05	27322.62	14.60	155.05	27322.62	26.63	282.82	49837.15
2037	14.60	155.05	27322.62	14.60	155.05	27322.62	27.15	288.37	50816.05
2038	14.60	155.05	27322.62	14.60	155.05	27322.62	27.68	293.93	51794.94
2039	14.60	155.05	27322.62	14.60	155.05	27322.62	28.20	299.48	52773.83
2040	14.60	155.05	27322.62	14.60	155.05	27322.62	28.72	305.04	53752.72
2041	14.60	155.05	27322.62	14.60	155.05	27322.62	29.25	310.59	54731.62
2042	14.60	155.05	27322.62	14.60	155.05	27322.62	29.77	316.15	55710.51
2043	14.60	155.05	27322.62	14.60	155.05	27322.62	30.29	321.70	56689.40
2044	14.60	155.05	27322.62	14.60	155.05	27322.62	30.82	327.26	57668.29
2045	14.60	155.05	27322.62	14.60	155.05	27322.62	31.34	332.81	58647.19
2046	14.60	155.05	27322.62	14.60	155.05	27322.62	31.86	338.37	59626.08
2047	14.60	155.05	27322.62	14.60	155.05	27322.62	32.38	343.92	60604.97
2048	14.60	155.05	27322.62	14.60	155.05	27322.62	32.91	349.48	61583.86
2049	14.60	155.05	27322.62	14.60	155.05	27322.62	33.43	355.03	62562.76
2050	14.60	155.05	27322.62	14.60	155.05	27322.62	33.95	360.59	63541.65
2051	14.60	155.05	27322.62	14.60	155.05	27322.62	34.48	366.14	64520.54
2052	14.60	155.05	27322.62	14.60	155.05	27322.62	35.00	371.70	65499.44

1.3 Total Carbon in the forest

Table S5: Total carbon stocks in the forest (from tables S1, S2,S3 & S4). All values are given in mio kg carbon

	BL			WPS			NPS		
Year	Trees	Deadwood	Total	Trees	Deadwood	Total	Trees	Deadwood	Total
2013	1037200.00	27322.62	1064522.62	1037200.00	27322.62	1064522.62	1037200.00	27322.62	1064522.62
2014	1036442.94	27322.62	1063765.57	1026217.00	27322.62	1053539.62	1037570.00	27322.62	1064892.62
2015	1035685.89	27322.62	1063008.51	1015234.00	27322.62	1042556.62	1037940.00	27322.62	1065262.62
2016	1034928.83	27322.62	1062251.45	1004251.00	27322.62	1031573.62	1038310.00	27322.62	1065632.62
2017	1034171.77	27322.62	1061494.40	993268.00	27322.62	1020590.62	1038680.00	27322.62	1066002.62
2018	1033414.72	27322.62	1060737.34	982285.00	27322.62	1009607.62	1039050.00	27322.62	1066372.62
2019	1032708.34	27322.62	1060030.96	969841.50	27322.62	997164.12	1039790.00	27322.62	1067112.62
2020	1032001.96	27322.62	1059324.58	957398.00	27322.62	984720.62	1040530.00	27322.62	1067852.62
2021	1031295.58	27322.62	1058618.20	944954.50	27322.62	972277.12	1041270.00	27322.62	1068592.62
2022	1030589.20	27322.62	1057911.82	932511.00	27322.62	959833.62	1042010.00	27322.62	1069332.62
2023	1029882.82	27322.62	1057205.44	920067.50	27322.62	947390.12	1042750.00	27322.62	1070072.62
2024	1033905.30	27322.62	1061227.92	911408.50	27322.62	938731.12	1046520.00	27322.62	1073842.62
2025	1037927.78	27322.62	1065250.40	902749.50	27322.62	930072.12	1050290.00	27322.62	1077612.62
2026	1041950.25	27322.62	1069272.88	894090.50	27322.62	921413.12	1054060.00	27322.62	1081382.62
2027	1045972.73	27322.62	1073295.35	885431.50	27322.62	912754.12	1057830.00	27322.62	1085152.62
2028	1049995.21	27322.62	1077317.83	876772.50	27322.62	904095.12	1061600.00	27322.62	1088922.62
2029	1052520.24	27322.62	1079842.86	867859.50	27322.62	895182.12	1066050.00	27322.62	1093372.62
2030	1055045.28	27322.62	1082367.90	858946.50	27322.62	886269.12	1070500.00	27322.62	1097822.62
2031	1057570.31	27322.62	1084892.93	850033.50	27322.62	877356.12	1074950.00	27322.62	1102272.62
2032	1060095.34	27322.62	1087417.97	841120.50	27322.62	868443.12	1079400.00	27322.62	1106722.62
2033	1062620.38	27322.62	1089943.00	832207.50	27322.62	859530.12	1083850.00	27322.62	1111172.62
2034	1065364.18	27322.62	1092686.80	830277.50	27322.62	857600.12	1087620.00	27322.62	1114942.62
2035	1068107.99	27322.62	1095430.61	828347.50	27322.62	855670.12	1091390.00	27322.62	1118712.62
2036	1070851.79	27322.62	1098174.41	826417.50	27322.62	853740.12	1095160.00	27322.62	1122482.62
2037	1073595.60	27322.62	1100918.22	824487.50	27322.62	851810.12	1098930.00	27322.62	1126252.62
2038	1076339.40	27322.62	1103662.02	822557.50	27322.62	849880.12	1102700.00	27322.62	1130022.62
2039	1078287.97	27322.62	1105610.59	822520.50	27322.62	849843.12	1106655.00	27322.62	1133977.62
2040	1080236.54	27322.62	1107559.16	822483.50	27322.62	849806.12	1110610.00	27322.62	1137932.62
2041	1082185.10	27322.62	1109507.73	822446.50	27322.62	849769.12	1114565.00	27322.62	1141887.62
2042	1084133.67	27322.62	1111456.29	822409.50	27322.62	849732.12	1118520.00	27322.62	1145842.62
2043	1086082.24	27322.62	1113404.86	822372.50	27322.62	849695.12	1122475.00	27322.62	1149797.62
2044	1087874.18	27322.62	1115196.80	826189.00	27322.62	853511.62	1125750.00	27322.62	1153072.62
2045	1089666.12	27322.62	1116988.74	830005.50	27322.62	857328.12	1129025.00	27322.62	1156347.62
2046	1091458.06	27322.62	1118780.69	833822.00	27322.62	861144.62	1132300.00	27322.62	1159622.62
2047	1093250.00	27322.62	1120572.63	837638.50	27322.62	864961.12	1135575.00	27322.62	1162897.62
2048	1095041.94	27322.62	1122364.57	841455.00	27322.62	868777.62	1138850.00	27322.62	1166172.62
2049	1095746.32	27322.62	1123068.94	846539.50	27322.62	873862.12	1143300.00	27322.62	1170622.62
2050	1096450.70	27322.62	1123773.32	851624.00	27322.62	878946.62	1147750.00	27322.62	1175072.62
2051	1097155.08	27322.62	1124477.70	856708.50	27322.62	884031.12	1152200.00	27322.62	1179522.62
2052	1097859.46	27322.62	1125182.08	861793.00	27322.62	889115.62	1156650.00	27322.62	1183972.62

2 Extracted carbon in raw wood

The carbon extracted in raw wood was calculated based on the annual raw wood potential, as well as tree species-specific carbon contents. The annual raw wood potential of the two scenarios was taken from Oehmichen et al. (2018) (Figure 2 and Figure 24, respectively). According to the methodology presented there, reduced raw wood potentials were calculated from the raw wood potential after deduction of dead wood resupply. The carbon content for the calculation of the contained carbon were calculated based on densities for the different tree species. The values for densities were taken from the wood database of the University of Dresden (Technische Universität Dresden 2017) and are shown in table S6.

Table S6: Conversion factors for carbon in different tree species groups. Raw densities of tree species were taken from the wood database of Technische Universität Dresden (2017). For the raw density of the wood species, the mean of the respective tree species was calculated to maintain coherence between stocks and raw wood potential.

Wood / Species Group	Density [t/m³]	Carbon Content [tC/t Wood]	Convernsion Factor [tC / m³]
Beech	0.690	0.5	0.345
Spruce	0.470	0.5	0.235
Pine	0.520	0.5	0.260
Oak	0.670	0.5	0.335
Hardwood	0.680	0.5	0.340
Softwood	0.495	0.5	0.2475

Table S7: Wood and carbon volumes extracted in raw wood of the **BL**. For each tree species, the respective carbon factor (C-factor) was used to calculate carbon masses from the wood volumes. In the last column the resulting total carbon masses are given

	Softwood		Hardwood		Total
C-Factor [kg/m^3]	247.5		340		-
Year	Wood mio m^3	C mio kg	Wood mio m^3	C mio kg	C mio kg
2013	51.26	12687.38	33.66	11443.37	24130.74
2014	51.26	12687.38	33.66	11443.37	24130.74
2015	51.26	12687.38	33.66	11443.37	24130.74
2016	51.26	12687.38	33.66	11443.37	24130.74
2017	51.26	12687.38	33.66	11443.37	24130.74
2018	51.00	12623.30	31.07	10563.11	23186.41
2019	51.00	12623.30	31.07	10563.11	23186.41
2020	51.00	12623.30	31.07	10563.11	23186.41
2021	51.00	12623.30	31.07	10563.11	23186.41
2022	51.00	12623.30	31.07	10563.11	23186.41
2023	46.34	11469.90	25.89	8802.59	20272.49
2024	46.34	11469.90	25.89	8802.59	20272.49
2025	46.34	11469.90	25.89	8802.59	20272.49
2026	46.34	11469.90	25.89	8802.59	20272.49
2027	46.34	11469.90	25.89	8802.59	20272.49
2028	47.64	11790.29	27.31	9286.73	21077.02
2029	47.64	11790.29	27.31	9286.73	21077.02
2030	47.64	11790.29	27.31	9286.73	21077.02
2031	47.64	11790.29	27.31	9286.73	21077.02
2032	47.64	11790.29	27.31	9286.73	21077.02
2033	47.90	11854.37	26.41	8978.64	20833.01
2034	47.90	11854.37	26.41	8978.64	20833.01
2035	47.90	11854.37	26.41	8978.64	20833.01
2036	47.90	11854.37	26.41	8978.64	20833.01
2037	47.90	11854.37	26.41	8978.64	20833.01
2038	48.16	11918.45	26.54	9022.65	20941.10
2039	48.16	11918.45	26.54	9022.65	20941.10
2040	48.16	11918.45	26.54	9022.65	20941.10
2041	48.16	11918.45	26.54	9022.65	20941.10
2042	48.16	11918.45	26.54	9022.65	20941.10
2043	48.41	11982.52	26.54	9022.65	21005.18
2044	48.41	11982.52	26.54	9022.65	21005.18
2045	48.41	11982.52	26.54	9022.65	21005.18
2046	48.41	11982.52	26.54	9022.65	21005.18
2047	48.41	11982.52	26.54	9022.65	21005.18
2048	51.26	12687.38	26.41	8978.64	21666.02
2049	51.26	12687.38	26.41	8978.64	21666.02
2050	51.26	12687.38	26.41	8978.64	21666.02
2051	51.26	12687.38	26.41	8978.64	21666.02
2052	51.26	12687.38	26.41	8978.64	21666.02

Table S8: Wood and carbon volumes extracted in raw wood of the **WPS**. For each tree species, the respective carbon factor (C-factor) was used to calculate carbon masses from the wood volumes. In the last column the resulting total carbon masses are given

	Spruce		Beech		Pine		Oak		Total
C-Factor [kg/m^3]	235		345		260		335		-
Year	Wood mio m^3	C mio kg	Wood mio m^3	C mio kg	Wood mio m^3	C mio kg	Wood mio m^3	C mio kg	C mio kg
2013	42.86	10071.82	34.15	11783.33	21.86	5682.34	9.27	3106.08	30643.56
2014	42.86	10071.82	34.15	11783.33	21.86	5682.34	9.27	3106.08	30643.56
2015	42.86	10071.82	34.15	11783.33	21.86	5682.34	9.27	3106.08	30643.56
2016	42.86	10071.82	34.15	11783.33	21.86	5682.34	9.27	3106.08	30643.56
2017	42.86	10071.82	34.15	11783.33	21.86	5682.34	9.27	3106.08	30643.56
2018	45.69	10737.73	32.89	11348.70	24.08	6260.44	9.57	3207.48	31554.34
2019	45.69	10737.73	32.89	11348.70	24.08	6260.44	9.57	3207.48	31554.34
2020	45.69	10737.73	32.89	11348.70	24.08	6260.44	9.57	3207.48	31554.34
2021	45.69	10737.73	32.89	11348.70	24.08	6260.44	9.57	3207.48	31554.34
2022	45.69	10737.73	32.89	11348.70	24.08	6260.44	9.57	3207.48	31554.34
2023	45.22	10627.66	30.46	10510.34	23.65	6149.72	8.80	2947.61	30235.32
2024	45.22	10627.66	30.46	10510.34	23.65	6149.72	8.80	2947.61	30235.32
2025	45.22	10627.66	30.46	10510.34	23.65	6149.72	8.80	2947.61	30235.32
2026	45.22	10627.66	30.46	10510.34	23.65	6149.72	8.80	2947.61	30235.32
2027	45.22	10627.66	30.46	10510.34	23.65	6149.72	8.80	2947.61	30235.32
2028	47.49	11160.48	30.14	10399.77	26.16	6802.36	8.34	2794.50	31157.12
2029	47.49	11160.48	30.14	10399.77	26.16	6802.36	8.34	2794.50	31157.12
2030	47.49	11160.48	30.14	10399.77	26.16	6802.36	8.34	2794.50	31157.12
2031	47.49	11160.48	30.14	10399.77	26.16	6802.36	8.34	2794.50	31157.12
2032	47.49	11160.48	30.14	10399.77	26.16	6802.36	8.34	2794.50	31157.12
2033	42.14	9902.87	24.50	8450.97	20.74	5392.78	6.66	2232.29	25978.90
2034	42.14	9902.87	24.50	8450.97	20.74	5392.78	6.66	2232.29	25978.90
2035	42.14	9902.87	24.50	8450.97	20.74	5392.78	6.66	2232.29	25978.90
2036	42.14	9902.87	24.50	8450.97	20.74	5392.78	6.66	2232.29	25978.90
2037	42.14	9902.87	24.50	8450.97	20.74	5392.78	6.66	2232.29	25978.90
2038	42.55	9999.79	23.71	8181.00	19.12	4971.31	6.65	2229.31	25381.41
2039	42.55	9999.79	23.71	8181.00	19.12	4971.31	6.65	2229.31	25381.41
2040	42.55	9999.79	23.71	8181.00	19.12	4971.31	6.65	2229.31	25381.41
2041	42.55	9999.79	23.71	8181.00	19.12	4971.31	6.65	2229.31	25381.41
2042	42.55	9999.79	23.71	8181.00	19.12	4971.31	6.65	2229.31	25381.41
2043	38.97	9158.07	21.11	7283.95	16.28	4231.88	5.58	1869.47	22543.38
2044	38.97	9158.07	21.11	7283.95	16.28	4231.88	5.58	1869.47	22543.38
2045	38.97	9158.07	21.11	7283.95	16.28	4231.88	5.58	1869.47	22543.38
2046	38.97	9158.07	21.11	7283.95	16.28	4231.88	5.58	1869.47	22543.38
2047	38.97	9158.07	21.11	7283.95	16.28	4231.88	5.58	1869.47	22543.38
2048	39.56	9297.30	20.34	7016.86	14.95	3887.57	5.39	1804.48	22006.21
2049	39.56	9297.30	20.34	7016.86	14.95	3887.57	5.39	1804.48	22006.21
2050	39.56	9297.30	20.34	7016.86	14.95	3887.57	5.39	1804.48	22006.21
2051	39.56	9297.30	20.34	7016.86	14.95	3887.57	5.39	1804.48	22006.21
2052	39.56	9297.30	20.34	7016.86	14.95	3887.57	5.39	1804.48	22006.21

3 Detailed Equations for RDF calculation

By inserting equation 8 of the main text into equation 9 of the main text we get:

$$\begin{aligned} DLCA(\Delta CF_{IF,y,t=y} - CW_{IF,y,t=y} + CW_{IF,y,t=y+z} - CW_{IF,y,t=y} * DF_y) * \frac{44}{12} \\ = DLCA(\Delta CF_{EF,y,t=y} - CW_{EF,y,t=y} + CW_{EF,y,t=y+z} - CW_{EF,y,t=y} * DF_y) * \frac{44}{12} \end{aligned} \tag{1}$$

The global warming potential calculated with Dynamic LCA provides the same result for the sum of several emissions as the addition of the individual global warming potentials calculated in this way. Therefore, the associative law applies here:

$$\begin{aligned} DLCA(\Delta CF_{IF,y,t=y}) - DLCA(CW_{IF,y,t=y}) + DLCA(CW_{IF,y,t=y+z}) \\ -DLCA(CW_{IF,y,t=y}) * DF_y \\ = DLCA(\Delta CF_{EF,y,t=y}) - DLCA(CW_{EF,y,t=y}) + DLCA(CW_{EF,y,t=y+z}) \\ -DLCA(CW_{EF,y,t=y}) * DF_y \end{aligned} \tag{2}$$

By simplifying we get:

$$\begin{aligned} DLCA(\Delta CF_{IF,y,t=y} - \Delta CF_{EF,y,t=y} - CW_{IF,y,t=y} + CW_{EF,y,t=y} \\ +CW_{IF,y,t=y+z} - CW_{EF,y,t=y+z}) \\ = DF_y * DLCA(-CW_{EF,y,t=y} + CW_{IF,y,t=y}) \end{aligned} \tag{3}$$

Dividing this equation by $DLCA(-CW_{EF,y,t=y}+CW_{IF,y,t=y}$ yields equation 10 in the main text.

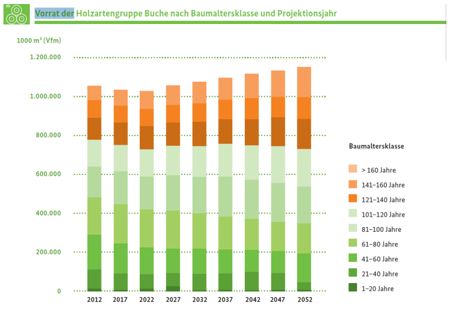

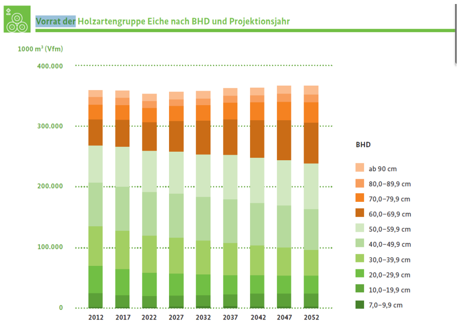

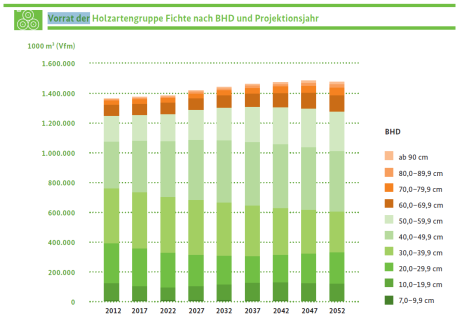

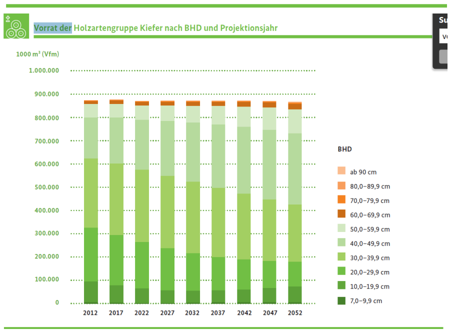

Abbildung 22: Entwicklung der Holzvorräte für Laub- und Nadelbäume zwischen 2012 und 2052

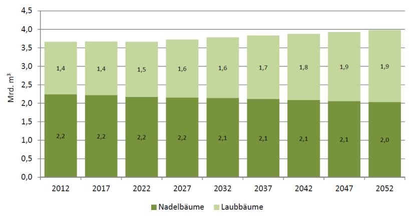

Figure S1: Wood stocks in BL (1-4) and NPS (5) from BMEL (2016) and Oehmichen et al. (2018).

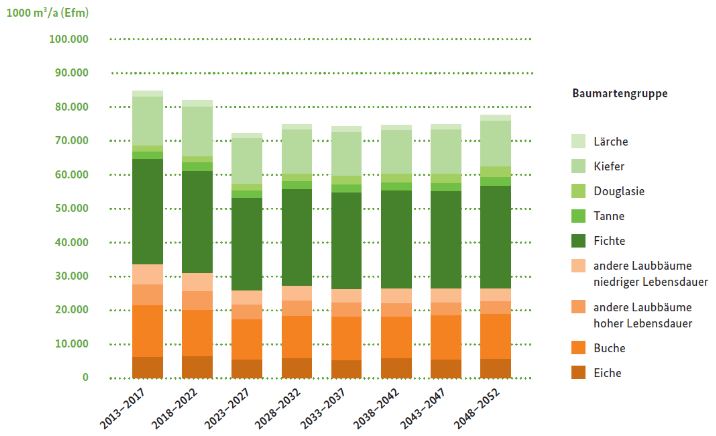

Figure S2: Raw wood extractions in BL (from BMEL (2016)).

Table S9: Wood and carbon volumes extracted in raw wood of the **NPS**. For each tree species, the respective carbon factor (C-factor) was used to calculate carbon masses from the wood volumes. In the last column the resulting total carbon masses are given

	Spruce		Beech		Pine		Oak		Total
C-Factor [kg/m^3]	235		345		260		335		-
Year	Wood mio m^3	C mio kg	Wood mio m^3	C mio kg	Wood mio m^3	C mio kg	Wood mio m^3	C mio kg	C mio kg
2013	28.08	6599.39	17.99	6204.98	17.28	4491.63	4.50	1506.28	18802.27
2014	28.08	6599.39	17.99	6204.98	17.28	4491.63	4.50	1506.28	18802.27
2015	28.08	6599.39	17.99	6204.98	17.28	4491.63	4.50	1506.28	18802.27
2016	28.08	6599.39	17.99	6204.98	17.28	4491.63	4.50	1506.28	18802.27
2017	28.08	6599.39	17.99	6204.98	17.28	4491.63	4.50	1506.28	18802.27
2018	29.96	7041.48	17.28	5961.83	17.99	4678.47	4.20	1407.42	19089.21
2019	29.96	7041.48	17.28	5961.83	17.99	4678.47	4.20	1407.42	19089.21
2020	29.96	7041.48	17.28	5961.83	17.99	4678.47	4.20	1407.42	19089.21
2021	29.96	7041.48	17.28	5961.83	17.99	4678.47	4.20	1407.42	19089.21
2022	29.96	7041.48	17.28	5961.83	17.99	4678.47	4.20	1407.42	19089.21
2023	27.39	6435.65	15.59	5379.79	16.14	4195.53	3.72	1247.49	17258.45
2024	27.39	6435.65	15.59	5379.79	16.14	4195.53	3.72	1247.49	17258.45
2025	27.39	6435.65	15.59	5379.79	16.14	4195.53	3.72	1247.49	17258.45
2026	27.39	6435.65	15.59	5379.79	16.14	4195.53	3.72	1247.49	17258.45
2027	27.39	6435.65	15.59	5379.79	16.14	4195.53	3.72	1247.49	17258.45
2028	26.68	6268.95	16.04	5532.65	14.80	3848.80	3.62	1213.93	16864.33
2029	26.68	6268.95	16.04	5532.65	14.80	3848.80	3.62	1213.93	16864.33
2030	26.68	6268.95	16.04	5532.65	14.80	3848.80	3.62	1213.93	16864.33
2031	26.68	6268.95	16.04	5532.65	14.80	3848.80	3.62	1213.93	16864.33
2032	26.68	6268.95	16.04	5532.65	14.80	3848.80	3.62	1213.93	16864.33
2033	27.15	6380.05	15.66	5401.67	14.81	3850.24	3.63	1214.38	16846.34
2034	27.15	6380.05	15.66	5401.67	14.81	3850.24	3.63	1214.38	16846.34
2035	27.15	6380.05	15.66	5401.67	14.81	3850.24	3.63	1214.38	16846.34
2036	27.15	6380.05	15.66	5401.67	14.81	3850.24	3.63	1214.38	16846.34
2037	27.15	6380.05	15.66	5401.67	14.81	3850.24	3.63	1214.38	16846.34
2038	26.38	6198.16	16.53	5703.72	13.92	3618.70	3.61	1210.72	16731.29
2039	26.38	6198.16	16.53	5703.72	13.92	3618.70	3.61	1210.72	16731.29
2040	26.38	6198.16	16.53	5703.72	13.92	3618.70	3.61	1210.72	16731.29
2041	26.38	6198.16	16.53	5703.72	13.92	3618.70	3.61	1210.72	16731.29
2042	26.38	6198.16	16.53	5703.72	13.92	3618.70	3.61	1210.72	16731.29
2043	26.20	6156.99	16.42	5665.83	12.45	3237.18	3.67	1228.27	16288.26
2044	26.20	6156.99	16.42	5665.83	12.45	3237.18	3.67	1228.27	16288.26
2045	26.20	6156.99	16.42	5665.83	12.45	3237.18	3.67	1228.27	16288.26
2046	26.20	6156.99	16.42	5665.83	12.45	3237.18	3.67	1228.27	16288.26
2047	26.20	6156.99	16.42	5665.83	12.45	3237.18	3.67	1228.27	16288.26
2048	25.21	5924.71	17.52	6043.91	11.65	3029.95	3.66	1224.78	16223.34
2049	25.21	5924.71	17.52	6043.91	11.65	3029.95	3.66	1224.78	16223.34
2050	25.21	5924.71	17.52	6043.91	11.65	3029.95	3.66	1224.78	16223.34
2051	25.21	5924.71	17.52	6043.91	11.65	3029.95	3.66	1224.78	16223.34
2052	25.21	5924.71	17.52	6043.91	11.65	3029.95	3.66	1224.78	16223.34

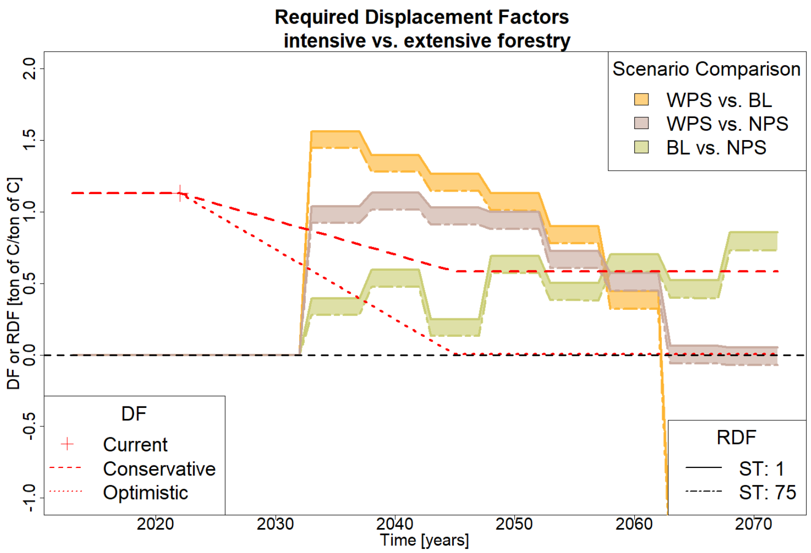

Figure S3: Required displacement factors in a thought experiment where a change in forest management starts in 2032

4 Thought experiment: Late change in forest management

Which of the forest management scenarios is preferable from a climate perspective depends on the trajectory of RDF and DF curves. In the analysis of our work DFs were comparably high at the beginning of the modeling period but decline over time. Precisely in that early period high DFs are also needed for intensive forestry to compensate for declining carbon stocks in the forest, leading to relatively high RDFs. Hence, intensive forestry seemed a feasible mean to combat climate change. But how would the result look like, if logging was intensified much later, i.e. in a time with relatively low DF values ? For that, we conducted a thought experiment where the current status of the forest would not change during the next 20 years and the WEHAM scenarios apply for the time between 2032 and 2072. The DF trajectory was not altered. Figure S3 and S4 show the result of this thought experiment. It can be seen, that RDF are much higher than DF for most of the time in the comparisons were the WPS is included. As a result, the BL and NPS perform better than the WPS regarding climate change mitigation. The cumulative GWP differences prove this conclusion as well. Whether BL or NPS performs better depends on DF estimates. The main conclusion drawn from this is the fact that from a climate perspective an intensification of logging should be initiated in a time where DFs are still high. Otherwise more extensive forest management practices have lower impact on climate change.

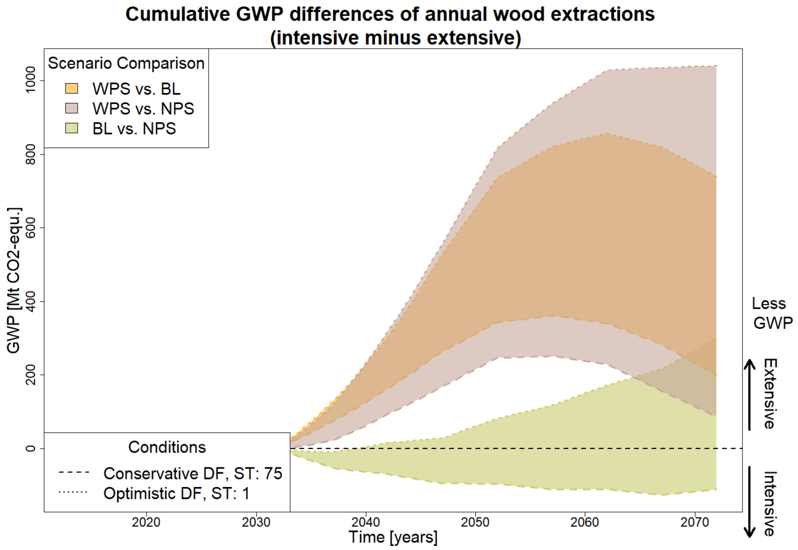

Figure S4: Cumulative GWP differences in a thought experiment where a change in forest management starts in 2032
